# Dorsoventral Patterning of the Mouse Coat by *Tbx15*

**DOI:** 10.1371/journal.pbio.0020003

**Published:** 2004-01-20

**Authors:** Sophie I Candille, Catherine D. Van Raamsdonk, Changyou Chen, Sanne Kuijper, Yanru Chen-Tsai, Andreas Russ, Frits Meijlink, Gregory S Barsh

**Affiliations:** **1**Departments of Genetics and Pediatrics, Stanford University School of MedicineStanford, CaliforniaUnited States of America; **2**Netherlands Institute for Developmental BiologyUtrechtThe Netherlands; **3**Genetics Unit, Department of BiochemistryUniversity of Oxford, OxfordUnited Kingdom

## Abstract

Many members of the animal kingdom display coat or skin color differences along their dorsoventral axis. To determine the mechanisms that control regional differences in pigmentation, we have studied how a classical mouse mutation, *droopy ear* (*de^H^*), affects dorsoventral skin characteristics, especially those under control of the *Agouti* gene. Mice carrying the *Agouti* allele *black-and-tan* (*a^t^*) normally have a sharp boundary between dorsal black hair and yellow ventral hair; the *de^H^* mutation raises the pigmentation boundary, producing an apparent dorsal-to-ventral transformation. We identify a 216 kb deletion in *de^H^* that removes all but the first exon of the *Tbx15* gene, whose embryonic expression in developing mesenchyme correlates with pigmentary and skeletal malformations observed in *de^H^*/*de^H^* animals. Construction of a targeted allele of *Tbx15* confirmed that the *de^H^* phenotype was caused by *Tbx15* loss of function. Early embryonic expression of *Tbx15* in dorsal mesenchyme is complementary to *Agouti* expression in ventral mesenchyme; in the absence of *Tbx15*, expression of *Agouti* in both embryos and postnatal animals is displaced dorsally. Transplantation experiments demonstrate that positional identity of the skin with regard to dorsoventral pigmentation differences is acquired by E12.5, which is shortly after early embryonic expression of *Tbx15*. Fate-mapping studies show that the dorsoventral pigmentation boundary is not in register with a previously identified dermal cell lineage boundary, but rather with the limb dorsoventral boundary. Embryonic expression of *Tbx15* in dorsolateral mesenchyme provides an instructional cue required to establish the future positional identity of dorsal dermis. These findings represent a novel role for T-box gene action in embryonic development, identify a previously unappreciated aspect of dorsoventral patterning that is widely represented in furred mammals, and provide insight into the mechanisms that underlie region-specific differences in body morphology.

## Abstract 2

Greg Barsh and colleagues show that a member of the well-known family of T-box genes helps to form an important pigmentation boundary in mice

## Introduction

A fundamental question in developmental biology is how adjacent regions of the vertebrate body acquire differences in their appearance or morphology. Mechanisms that establish the general body plan make use of a relatively small number of signaling pathways shared among all animals (reviewed in Pires-daSilva and Sommer 2003), but the extent to which these pathways control finer differences between body regions is not clear. Among vertebrates, differences in the shape or number of skeletal elements, altered morphology of epidermal appendages, and variation in pigment distribution combine to produce the majority of what distinguishes one animal from another. Among these, pigment patterns are an excellent system to investigate how morphological differences arise, both for different regions of the body within a species and for different animals from closely related species. In natural environments, color variation is a nearly universal mechanism for recognition, camouflage, or both; consequently, a large number of pigment patterns have been characterized from an evolutionary and ecological perspective (Boughman 2001; Jiggins et al. 2001). In the laboratory, color variation has been the subject of vertebrate genetics for more than a century (Searle 1968; Silvers 1979), and many pigmentary components have been identified whose actions are understood in a cellular or organ-based context (reviewed in Bennett and Lamoreux 2003).

Several mechanisms may contribute to regional differences in vertebrate pigmentation. In the embryo, alterations in the determination or migration of melanoblasts from the neural crest affect the number or distribution of pigment cells in the skin (reviewed in Reedy et al. 1998). Within hair follicles, paracrine signals control the type of pigment made in specific regions of the body or at specific times during the hair cycle (reviewed in Furumura et al. 1996; Barsh et al. 2000). Finally, movement of pigment granules within melanocytes or from melanocytes to keratinocytes makes use of cellular machinery that is shared by a variety of cell types, but that can vary in different regions of the body (reviewed in Marks and Seabra 2001). However, for all of these mechanisms—white spotting, pigment-type switching, and melanosome biogenesis—more is known about the identity of the molecular components than their spatial and temporal control.

One of the most obvious aspects of regional color variation in vertebrates is a dark dorsal surface juxtaposed to a light ventral surface, apparent in the color of skin, scales, feathers, or hair, in which the boundary between dorsal and ventral compartments is often sharp and lies in register with the limbs. In rodents and probably other mammals, this dorsoventral difference in hair color is brought about by differences in pigment type as determined by allelic variation of the *Agouti* gene (Bultman et al. 1992; Miller et al. 1993). Secreted by dermal papilla cells within each hair follicle (Millar et al. 1995), Agouti protein causes melanocytes in that follicle to switch from the production of brown/black eumelanin to red/yellow pheomelanin. Agouti protein has a short radius of action (Silvers and Russel 1955) and can be switched on and off during a single hair cycle (Bultman et al. 1992, 1994; Miller et al. 1993; Vrieling et al. 1994); thus, its regulated expression is thought to be responsible for the cream-colored or yellow ventral surface of mice carrying the *black-and-tan* (*a^t^*) allele and for the yellow markings around the feet, ears, or head, i.e., tan points or head spots, of certain dog breeds.

In laboratory mice, previous studies from our group and others identified two predominant *Agouti* mRNA isoforms that differ by virtue of their transcriptional initiation site and 5′ untranslated exons. A “hair cycle-specific” transcript is expressed in both dorsal and ventral skin for 2–3 days during early hair growth, while a “ventral-specific” transcript is expressed throughout the entire period of active hair growth, but only in ventral skin (Bultman et al. 1994; Vrieling et al. 1994). Animals carrying the *a^t^* allele express only the ventral-specific *Agouti* transcript (Bultman et al. 1994; Vrieling et al. 1994) and have black dorsal hairs and cream-colored to yellow ventral hairs, with a sharp boundary at the level of the limb–body wall articulations and in the middle of the whisker pad. Ventral-specific *Agouti* isoforms are also expressed in developing skin from embryonic day 10.5 (E10.5) and beyond and may play a role in pigment cell differentiation (Millar et al. 1995). Thus, regulatory elements for ventral-specific *Agouti* isoforms are responsive to dorsoventral positional cues established in the embryo and whose effects persist after birth.

The boundary between dorsal and ventral color compartments in *a^t^*/*a^t^* mice bears superficial resemblance to dorsoventral boundaries apparent for many other mammals, but morphogenetic differences between dorsal and ventral skin seem likely to include more elements than the type of pigment made by hair follicle melanocytes. In particular, dermis of the flank has at least two distinct origins: dermatomal derivatives of somites and loose mesenchyme derived from the lateral plate mesoderm (Mauger 1972; Christ et al. 1983; Olivera-Martinez et al. 2000; Nowicki et al. 2003); these lineages are established early in development and could, in principle, set up compartments whose identity contributes to dorsoventral differences in adult skin.

To better understand the mechanisms that give rise to differences between dorsal and ventral skin and to the boundary between them, we have determined how several morphologic characteristics vary along the dorsoventral axis of the mouse and how these characteristics correspond to ventral-specific *Agouti* expression and the lineage boundary that distinguishes somite from lateral plate derivatives. Our results indicate that the apparent uniformity of the dorsoventral boundary represents the sum of independent mechanisms that affect melanocyte density and/or differentiation, pigment-type synthesis, and hair length; surprisingly, none of these coincide with the somite–lateral plate lineage boundary. We also make use of a classical mouse mutation, *droopy ear* (Curry 1959), that produces a dorsal-to-ventral transformation of flank coat color by allowing expansion of the ventral-specific *Agouti* transcript. By positional cloning and gene targeting, we identify an allele of *droopy ear*, *de^H^*, as a loss of function for *Tbx15*, which encodes a T-box transcription factor expressed in a dynamic and spatially restricted manner in the developing skin and musculoskeletal system. Embryonic expression and transplantation studies suggest that *Tbx15* is required to establish certain characteristics of dorsal patterning in mesenchymal cells of the developing flank. These results identify a previously unappreciated aspect of dorsoventral patterning that is widely represented in furred mammals and provide insight into the mechanisms that underlie region-specific differences in body morphology.

## Results

### Morphological Components of Dorsoventral Skin Differences

Besides the obvious change in hair color that frequently distinguishes dorsal from ventral skin, casual observation suggests there are additional differences in hair length, distribution of hair type, and skin thickness. Furthermore, dorsoventral differences in pigmentation can represent differences in the number and/or differentiated state of pigment cells, as well as the type of pigment synthesized in response to expression of *Agouti*. In particular, ventral hair of *a^t^*/*a^t^* animals can vary from cream-colored to reddish-yellow depending on age, strain background, and position along the dorsoventral axis. To evaluate the relationship among these components, we compared their features among mice of different *Agouti* genotypes.

**Figure 1:**
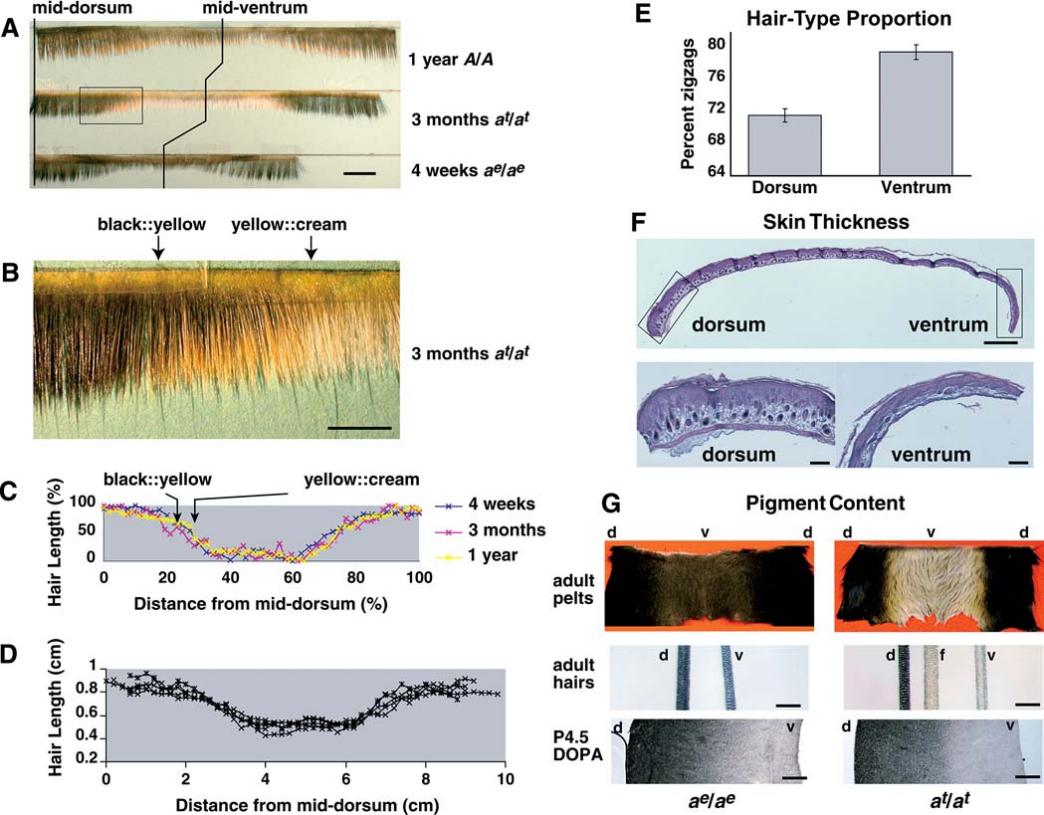
Dorsoventral Skin Characteristics (A) Skin slices from animals of different age and genotype demonstrate similar patterns of hair-length variation along the dorsoventral axis (scale bar = 1 cm). (B) Enlarged area from (A), demonstrating the transition in hair length and color in *a^t^*/*a^t^* mice (scale bar = 0.375 cm). (C) Proportional hair length for (A) plotted as a function of relative position along the dorsoventral axis. (D) Hair length plotted as a function of absolute position along the dorsoventral axis for 8-wk-old BA strain mice. (E) Proportion of zigzag hairs (± SEM) differs slightly between dorsum and ventrum of inbred mice (*p* < 0.0001, χ^2^ test, *n* = 1,958, 1,477, 1,579, 1,502). (F) Differences in dorsal and ventral skin development at P4.5 (scale bar = 1 mm, upper; 200 μm, lower). (G) Differences in hair melanin content and DOPA staining for dorsum (d), flank (f), and ventrum (v) in *a^e^*/*a^e^* and *a^t^*/*a^t^* mice. The upper panel also demonstrates a cream-colored appearance of the *a^t^*/*a^t^* ventrum. The middle panel shows representative awls (scale bar = 100 μm). The lower panel shows DOPA-stained dermis (scale bar = 200 μm).

Semiquantitative measurements of hair length plotted as a function of dorsoventral position reveal that the apparent sharp boundary between dorsal and ventral pigment compartments in *a^t^*/*a^t^* mice coincides with a more gradual change in both hair color and hair length (Figure 1A–1D). Within the region of transition from dorsum to ventrum (Figure 1B), flank hairs from *a^t^*/*a^t^* mice become progressively shorter and exhibit increasing amounts of pheomelanin deposition progressing from the tip to the base of the hair. However, the region of transition for hair length is considerably broader than that for pigmentation and independent of *Agouti* genotype. Although hair-cycle timing varies along the rostrocaudal axis, measurements of absolute hair length for mice matched for age and rostrocaudal level are remarkably similar (Figure 1D). Furthermore, measurements of relative hair length for animals of different age, size, and *Agouti* genotype also are very similar when normalized to body circumference (Figure 1C). Taken together, these observations indicate that variation of hair length along the dorsoventral axis is stereotyped and maintained through multiple hair cycles, with a transition in hair length that is gradual and encompasses the pigment-type transition in *a^t^*/*a^t^* mice.

Dorsal and ventral skin develop at different rates. Transverse sections of skin at postnatal day 4.5 (P4.5) exhibit dorsal hair follicles that are noticeably more developed than ventral hair follicles, along with a gradual dorsoventral decrease in dermal thickness (Figure 1F). However, differences in skin thickness disappear by 3–4 wk of age (Forsthoefel et al. 1966), and, overall, the proportion of different hair types is also similar in dorsa and ventra of adult mice. In age-matched inbred mice, we observed a small decrease in the ratio of undercoat hairs (zigzags) to overcoat hairs (auchenes, awls, and guard hairs) in dorsum compared to ventrum (Figure 1E), but there was no consistent difference in hair-type distribution for outbred mice (data not shown).

Differences between dorsal and ventral pigmentation of *a^t^*/*a^t^* mice are usually attributed to pigment-type differences caused by ventral-specific expression of *Agouti*, but animals homozygous for a null allele of *Agouti*, *extreme nonagouti* (*a^e^*), have ventral hairs that contain less melanin than dorsal hairs, giving a slightly paler appearance to the ventral coat (Figure 1G). Using DOPA staining as an indicator of tyrosinase activity, we observed a gradual dorsoventral transition in isolated dermis preparations from P4.5 *a^e^*/*a^e^* mice (Figure 1G). By contrast, skin from *a^t^*/*a^t^* mice reveal an abrupt dorsoventral transition of DOPA staining, which probably reflects the additive effects of reduced melanin content (as in *a^e^*/*a^e^* mice) and downregulation of tyrosinase activity induced by *Agouti*. Melanin content of individual hairs is likely to be influenced both by the number of pigment cells and their follicular environment. Regardless, dorsoventral differences in hair pigment content of *a^e^*/*a^e^* mice persist throughout multiple hair cycles into adulthood, similar to hair length (but unlike skin thickness). Thus, at least three characteristics distinguish dorsal from ventral skin: differences in pigment-type synthesis (depending on *Agouti* genotype), differences in hair length, and differences in melanin content.

### Ventralization of Skin Morphology by the *droopy ear* Mutation

Named after its effects on craniofacial morphology, *droopy ear* is a recessive mutation on mouse Chromosome 3; the original allele described more than 40 years ago by Curry (1959) is extinct, but a spontaneous remutation that occurred in Harwell, *de^H^*, is available through The Jackson Laboratory (Bar Harbor, Maine, United States). External craniofacial malformations are the most obvious characteristic of *de^H^*/*de^H^* animals, including widely spaced eyes, small palpebral fissures, a broad nasal area, and a shortened skull held in an elevated position, which presumably causes or contributes to the abnormal position of the ears.

We became interested in *droopy ear* because the original allele was described to affect pigment pattern in a way that suggests a possible dorsal to ventral transformation: “On a genetic background (*a^t^* and *A^W^*) which causes the belly hair to be lighter than the back hair, the belly hair comes up farther round the sides of the body and face” (Curry 1959).

**Figure 2:**
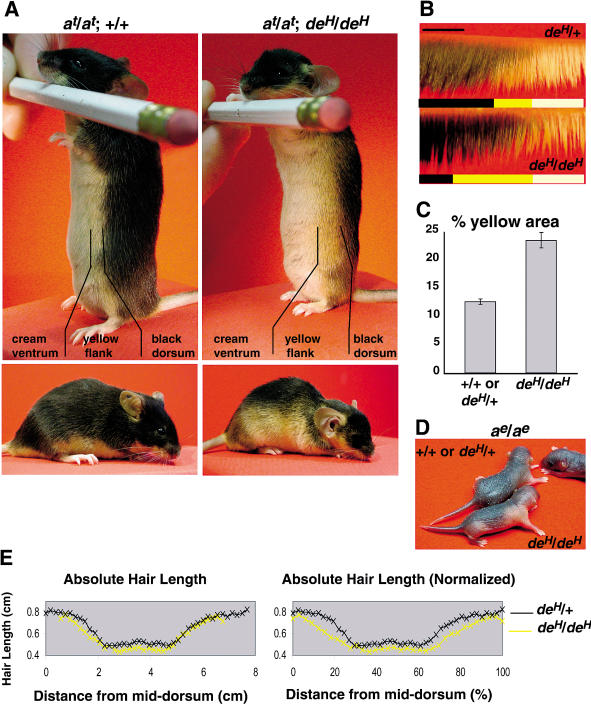
The *de^H^* Pigmentation Phenotype (A) 10-wk-old *de^H^/de^H^* and nonmutant animals on a *a^t^* background. A thin stripe of yellow hair normally separates the dorsal black hairs from the ventral cream hairs. In *de^H^*, the yellow stripe is extended dorsally, and the boundary between the yellow and the black hairs is fuzzier. (B) Skin slices taken from 1.5-mo-old *de^H^/de^H^* and nonmutant littermates (scale bar = 0.5 cm). (C) Proportion of total skin area as determined by observation of pelts taken from the interlimb region. The proportion occupied by the yellow lateral compartment (± SEM) differs between mutant and nonmutant littermate flanks (*p* < 0.0005, paired *t*-test, *n* = 6 pairs). There is also (data not shown) a small increase in the proportion of total skin area occupied by the ventral cream-colored compartment, 47.9 % in mutant compared to 37.8% in nonmutant (*p* < 0.005, paired *t*-test, *n* = 6 pairs). (D) On an *a^e^/a^e^* background, the extent of dorsal skin pigmentation is reduced in *de^H^/de^H^* neonates (P3.5). (E) Hair length in a representative pair of 1.5-mo-old *de^H^/de^H^* and nonmutant littermates, averaged over three skin slices at different rostrocaudal levels, and plotted as a function of the absolute distance from middorsum or the percentage of total slice length.

An abnormal dorsoventral pigment pattern is readily apparent in *a^t^*/*a^t^*; *de^H^*/*de^H^* mice, but comparison to nonmutant animals is more accurately described in terms of ventral, lateral, and dorsal regions (Figures 1G and 2A). The ventral region has short hairs with a gray base and cream-colored tip whose boundary coincides with the limb–body wall junction; both the appearance of this region and position of the boundary are approximately similar in *a^t^*/*a^t^* compared to *a^t^*/*a^t^*; *de^H^*/*de^H^* mice. The lateral region contains yellow hairs of progressively increasing length; in *a^t^*/*a^t^* mice, the lateral region appears as a thin yellow stripe along the flank, but in *a^t^*/*a^t^*; *de^H^*/*de^H^* mice, the lateral region is considerably expanded with a diffuse boundary along the dorsal flank, and a dorsal eumelanic region whose size is correspondingly reduced (Figure 2A and 2B). Total body size is smaller in mutant compared to nonmutant animals, but the proportion of body circumference occupied by the lateral region in mutant animals is increased about 2-fold, from 11.9% to 22.2% (Figure 2C). The proportion of the ventral cream-colored region is also expanded a small amount, 47.9% in mutant compared to 37.8% in nonmutant animals, but expansion of the lateral region, which occurs at all levels of the body, including the limbs and the cranium (but not the whisker pad), is the major feature responsible for the ventralized appearance caused by *de^H^*.

To investigate whether *de^H^* affects other dorsoventral skin characteristics besides pigment-type switching, we examined its effects on hair length and pigmentation in an *a^e^*/*a^e^* background. Overall, *de^H^* causes a small but consistent reduction in hair length in both dorsum and ventrum; when mutant and nonmutant animals are normalized for body circumference, reduced hair length is most apparent in the lateral region (Figure 2E). Adult *a^e^*/*a^e^*; *de^H^*/*de^H^* animals exhibit body-size reduction and skeletal abnormalities, but display no coat-color phenotype (data not shown). However, *a^e^*/*a^e^* and *a^e^*/*a^e^*; *de^H^*/*de^H^* neonates are clearly distinguishable in the first few days after birth, when a dorsoventral gradient of melanogenic activity is apparent under the skin (Figure 2D). At this stage, melanoblast migration from the neural crest is mostly complete, but there is a dorsoventral gradient in melanocyte differentiation and pigment synthesis. The skin of *a^e^*/*a^e^* neonates appears uniformly dark over the entire dorsum, but in *a^e^*/*a^e^*; *de^H^*/*de^H^* neonates, the area of dark skin is more restricted, particularly above the limbs, and resembles the pattern of dorsal eumelanin in *a^t^*/*a^t^*; *de^H^*/*de^H^* adult animals.

Taken together, these observations suggest that *de^H^* interferes with the establishment of dorsoventral patterning during skin development by causing dorsal expansion of a lateral region that is normally 3–5 mm in width. This same region may serve as a boundary between dorsal and ventral skin by inhibiting melanocyte differentiation, by promoting pheomelanin synthesis, and by supporting a progressive increase in hair growth from ventrum to dorsum. As described below, the gene defective in *de^H^*, *Tbx15*, is normally expressed in the dorsal region and therefore is likely to play a role in establishing the size and dorsal extent of the lateral region.

### Positional Cloning of *de^H^*

**Figure 3:**
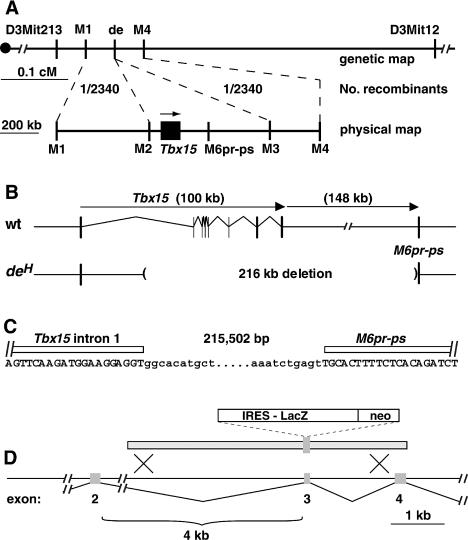
Molecular Genetics of *de^H^* and *Tbx15* (A) Genetic and physical map, as described in the text. Markers M1 to M3 are SSCP markers generated from a BAC contig of the region; marker M4 is STS 16.MMHAP32FLF1 and was also used as an SSCP marker. M2 and M3, which flank the *Tbx15* and *M6pr-ps* on the UCSC genome browser map and lie 634 kb apart, were nonrecombinant with *de^H^* in 2340 meioses. (B) The *de^H^* mutation is a deletion that starts in *Tbx15* intron 1 and ends in the *M6pr-ps*. (C) Sequence of deletion breakpoints. (D) Diagram of *Tbx15^LacZ^* allele constructed by gene targeting. As described in the text, this allele is predicted to give rise to a protein truncated after approximately 154 codons and is lacking critical residues of the T box. Heterozygotes for the targeted allele exhibit normal size, morphology, and hair-color patterns, but homozygotes and *Tbx15^LacZ^*/*de^H^* compound heterozygotes are identical to *de^H^* homozygotes.

As a visible marker, early linkage studies with the original *droopy ear* allele or the *de^H^* allele identified a map position in the middle of Chromosome 3, distal to *matted* and proximal to *Varitint-waddler* (Carter and Falconer 1951; Curry 1959; Lane and Eicher 1979; Holmes et al. 1981). We used an F_2_ intercross with CAST/Ei mice to localize *de^H^* to a 0.1 cM interval between D3Mit213 and 16.MMHAP32FLF1, which was refined by development of a bacterial artificial chromosome (BAC) contig and additional markers to a 1.4 Mb region that contained eight genes, including *Tbx15* (Figure 3A). We considered *Tbx15* as an excellent candidate for the skeletal abnormalities caused by *de^H^*, based on studies by Agulnik et al. (1998), who described its embryonic expression in the craniofacial region and developing limbs.

Using sequence information from Agulnik et al. (1998) and the partially completed mouse genome sequence, we found that portions of several *Tbx15* exons could not be amplified from *de^H^*/*de^H^* genomic DNA. The same gene was initially referred to as *Tbx8* (Wattler et al. 1998) and then later renamed *Tbx14*, but is currently referred to in several vertebrate genomes as *Tbx15* (Agulnik et al. 1998; Begemann et al. 2002). By comparing the sequence of a 1.3 kb junction fragment amplified from *de^H^*/*de^H^* genomic DNA to publicly available mouse genome sequence, we identified a 216 kb deletion that extends from *Tbx15* intron 1 to 148 kb downstream of the polyadenylation sequence in a region annotated as a mannose-6-phosphate receptor pseudogene, *M6pr-ps* (Figure 3B and 3C). (Ludwig et al. 1992). By Northern blot analysis, we identified a fusion transcript produced from the *de^H^* chromosome (data not shown). However, the deletion removes 534 of the 602 amino acids encoded by *Tbx15* (including the T-box DNA-binding domain), *de^H^*/+ animals are grossly normal, and the phenotype of *de^H^*/*de^H^* animals is identical to that described for the original allele. In addition, other than *M6pr-ps*, no other genes or transcripts have been annotated to the 216 kb deletion.

While the positional cloning work was underway, one of us (A. Russ) generated an independent mutation of *Tbx15* by gene targeting in embryonic stem cells. The targeted allele, *Tbx15^LacZ^*, carries an *IRES-LacZ-neo* cDNA cassette that disrupts the open reading frame at codon 154 early in the T-box domain (Figure 3D). Animals heterozygous for the targeted allele are completely normal with regard to size, skeletal morphology, and hair-color distribution, but *Tbx15^LacZ^*/*Tbx15^LacZ^* homozygotes were noted to exhibit reduced body size and an abnormal craniofacial appearance identical to that caused by *de^H^*. We generated *Tbx15^LacZ^*/*de^H^* compound heterozygotes; on an *A^w^*/*a^t^* background, these animals exhibited the same abnormal restriction of dorsal pigmentation at P3.5 and expanded yellow flank area as described above for *de^H^*/*de^H^* animals (see Figure 2). These observations demonstrate that the pigmentary and craniofacial characteristics of *de^H^* are caused by loss of function for *Tbx15*.

### Expression of *Tbx15* and *Agouti*

**Figure 4:**
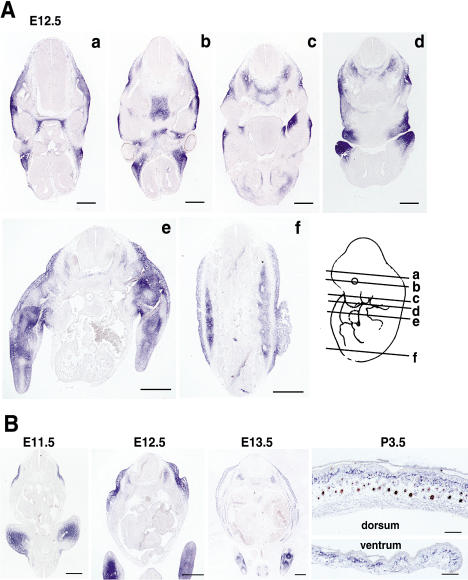
Developmental Expression of *Tbx15* (A) At E12.5, transverse sections at different levels show expression in head mesenchyme (a and b); myotome, occipital, and periocular mesenchyme (b); palatal shelf, cervical sclerotome, and nasal cartilage (c); maxillary and mandibular processes (d); limbs (e); and myotome and lateral mesenchyme (e and f) (scale bars = 500 μm). (B) Transverse sections through the flank at different times show expression in lateral mesenchyme (E11.5), expanding dorsally at E12.5, and both ventrally and dorsally at E13.5, detectable in loose mesenchyme underlying the dermis and the abdominal and subcutaneous muscles (scale bar = 500 μm). At P3.5, *Tbx15* is expressed in the entire dermis and is most strongly expressed in dermal sheaths (scale bar = 200 μm).

Previous studies by Agulnik et al. (1998) using whole-mount in situ hybridization described expression of *Tbx15* as first detectable at E9.5 in the limb buds, progressing to the branchial arches, flanks, and craniofacial regions through E12.5. To investigate this pattern in more detail, we hybridized a *Tbx15* mRNA probe to a series of transverse sections at E12.5 and observed expression in multiple mesenchymal tissues of the head, trunk, and developing limbs (Figure 4A), much of which is consistent with the skull, cervical vertebrae, and limb malformations reported for mice carrying the original *droopy ear* allele.

We were particularly interested in determining the exact nature of the embryonic flank expression relative to the ventralized phenotype of adult *de^H^*/*de^H^* mice. Transverse abdominal sections from different times during development reveal a dorsolateral band of expression in the superficial mesenchyme at E11.5 that broadens both dorsally and ventrally over the next several days (Figure 4B). By E13.5, the developing dermis has become separated from the loose mesenchyme by a subcutaneous muscle layer; *Tbx15* is expressed in all of these layers as well as the underlying abdominal muscles. In P3.5 skin, *Tbx15* is expressed in both dorsal and ventral skin, most strongly in the condensed upper dermis and developing dermal sheaths of hair follicles; faint expression can also be detected in rare dermal papillae cells (Figure 4B).

**Figure 5:**
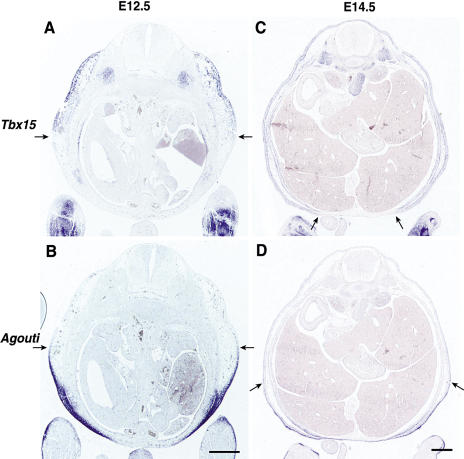
Embryonic Expression of *Tbx15* Compared to *Agouti* in *a^t^*/*a^t^* Mice (A and C) *Tbx15*. (B and D) *Agouti*. At E12.5, expression of *Tbx15* in dorsal skin is approximately complementary to that of *Agouti* in ventral skin. At E14.5, the levels of expression for both genes are lower, but *Tbx15* expression has expanded ventrally and overlaps extensively with that of *Agouti*. In all four panels, arrows mark the approximate ventral limit of *Tbx15* and the approximate dorsal limit of *Agouti* (scale bars = 500 μm).

Although the effects of *Agouti* on pigment-type switching occur during postnatal hair growth, the ventral-specific isoform of *Agouti* is expressed in developing skin beginning at E11.5. We compared adjacent sections hybridized with probes for *Tbx15* and *Agouti* and observed complementary patterns at E12.5, with expression of *Agouti* in ventral skin and expression of *Tbx15* in dorsal skin (Figure 5A and 5B). The junction between expression domains is indistinct, and by E14.5, *Tbx15* expression extends ventrally and overlaps extensively with *Agouti* expression (Figure 5C and 5D).

**Figure 6:**
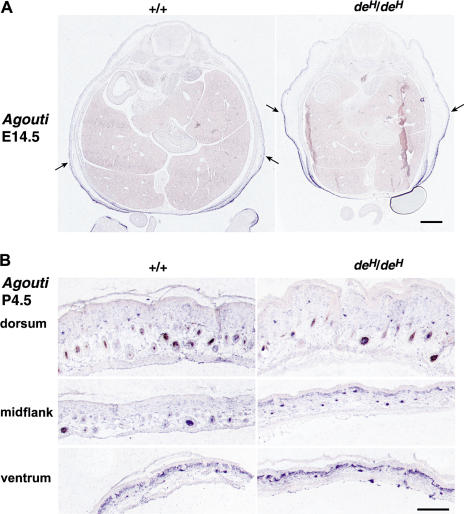
Effect of *de^H^* on *Agouti* Expression Comparable sections from *a^t^/a^t^*; *de^H^*/*de^H^* and *a^t^/a^t^*; +/+ littermates. (A) At E14.5, *de^H^*/*de^H^* embryos have a smaller body cavity and loose skin within which *Agouti* expression appears to be shifted dorsally, as marked by arrows (scale bars = 500 μm). (B) At P4.5, *Agouti* expression in both dorsal and ventral skin is similar in *de^H^*/*de^H^* compared to nonmutant, but in the midflank region, there is increased *Agouti* expression in *de^H^*/*de^H^*, especially in the upper dermis (scale bars = 200 μm). Sections shown are representative of two mutant and two nonmutant samples examined at each time.

We also examined the effect of *de^H^* on expression of *Agouti* and found no difference between mutant and nonmutant at E12.5 or E13.5 (data not shown). However, at E14.5, the normal ventral-to-dorsal gradient of *Agouti* expression appeared to extend more dorsally in *de^H^*/*de^H^* embryos (Figure 6A). In P4.5 skin, expression of *Agouti* is also extended dorsally in *de^H^*/*de^H^* animals and is most apparent in the midflank region within the upper dermis and dermal papillae cells (Figure 6B). Thus, while the pigmentation phenotype of *de^H^*/*de^H^* mice can be explained, not surprisingly, by dorsal extension of *Agouti* expression after birth, patterned expression of *Tbx15* and *Agouti* are apparent some 10 days earlier, between E12.5 and E13.5, and the effects of *Tbx15* deficiency on expression of *Agouti* can be detected by E14.5.

### Relationship of Embryonic *Tbx15* Expression to Dorsal and Ventral Pigmentation Domains

The observations described above are consistent with a model in which transient expression of *Tbx15* in the embryonic dorsal flank is required to establish positional identity of the future dermis, at least with respect to pigment-type synthesis caused by the ventral-specific *Agouti* isoform. To further investigate this hypothesis, we carried out transplantation experiments in which pieces of embryonic skin were isolated from different dorsoventral positions. We evaluated the embryonic skin fragments for their potential to give rise to different hair colors and for their expression of *Tbx15* and *Agouti*.

**Figure 7:**
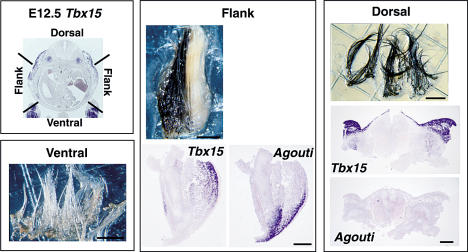
Embryonic Establishment of Dorsoventral Skin Patterning Pieces of skin from dorsal, flank, and ventral regions of *a^t^/a* E12.5 embryos were transplanted into the testes of congenic animals as described in the text. Hair color of the grafts was examined 3 wk later. Grafts of ventral embryonic skin (*n* = 3) produced yellow hairs, dorsal embryonic skin (*n* = 4) produced black hairs, and flank embryonic skin produced mostly (13 out of 15) black and yellow hairs in distinct regions as shown. In parallel, in situ hybridization studies revealed that the embryonic flank contains the boundary of expression between *Agouti* and *Tbx15* (scale bars = 1 mm for hairs and 200 μm for in situ hybridization results).

Previous studies by Silvers and colleagues (Poole and Silvers 1976) showed that dorsal and ventral skin isolated from *a^t^*/*a^t^* embryos gives rise to black and yellow hair, respectively, when transplanted into testis and allowed to develop for several weeks. Furthermore, dermal–epidermal recombination experiments carried out at E14.5 demonstrated that positional identity is carried by the embryonic dermis. In a variation on this experiment, we divided embryonic skin from *a^t^*/*a* embryos into dorsal, flank, and ventral pieces and analyzed the different pieces for their ability to give rise to black or yellow hair after testis transplantation, and, in parallel, for gene expression using in situ hybridization. For the purposes of a reproducible morphologic boundary, we divided flank from ventral skin based on a change in skin thickness and divided dorsal from flank skin at the level of an ectodermal notch that lies at the same level as the ventral extent of the myotome (Figure 7) (Huang and Christ 2000; Olivera-Martinez et al. 2000; Sudo et al. 2001; Burke and Nowicki 2003; Nowicki et al. 2003).

We found that E12.5 is the earliest time at which embryonic ventral skin is able to produce hair when transplanted to the testis. Of the grafts that produced hair, ventral skin gave rise to yellow hair (*n* = 3), and dorsal skin gave rise to black hair (*n* = 4). Transplantation of flank skin gave rise to a patch of yellow hair juxtaposed against a patch of black hair in 85% of the successful grafts (*n* = 13); the remaining two flank grafts produced solely black or yellow hair. In no case did we observe intermingling of black and yellow hairs. As predicted from the experiments using tissue sections (see Figures 5 and 6), dorsal pieces expressed *Tbx15* but not *Agouti*, while flank pieces expressed both genes (see Figure 7). Thus, dorsoventral identity for adult pigmentation is established by the time when patterned expression becomes apparent for *Tbx15* and *Agouti* (E11.5–E12.5); furthermore, positional identity is maintained throughout later stages of skin development, even though expression of *Tbx15* broadens to include ventral as well as dorsal skin.

### Relationship of the Dorsoventral Pigment Boundary to Lineage Compartments and the Lateral Somitic Frontier

The ectodermal notch that we used to mark the boundary between embryonic dorsum and embryonic flank is a characteristic feature in vertebrate embryos. In cell lineage studies carried out in the chick system, the notch serves as a landmark for the boundary between dermis derived from somitic mesoderm and dermis derived from lateral plate mesoderm and has been termed the “lateral somitic frontier” (Olivera-Martinez et al. 2000; Sudo et al. 2001; Burke and Nowicki 2003; Nowicki et al. 2003). Although fate-mapping studies have not been carried out in mammalian embryos, somite- and lateral plate-derived mesoderm could give rise to precursors for dermis dorsal and ventral to the limb–body wall junction, respectively. However, this notion conflicts with our observation that the future pigmentation boundary lies ventral to the ectodermal notch (see Figure 7).

**Figure 8:**
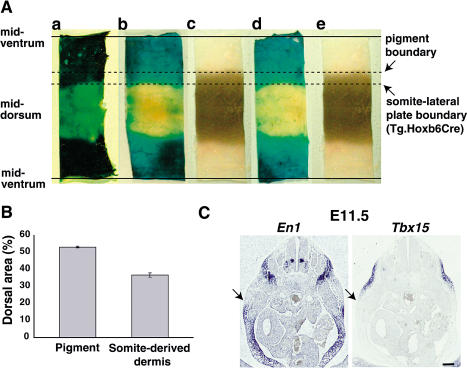
Comparison of the Dorsoventral *a^t^/a^t^* Pigmentation Boundary to the Lateral Somitic Frontier (A) Dorsoventral slices of skin from at the midtrunk region prepared such that the dorsal midline lies in the center of the slice. Sections were taken at P1.5 (a) or P4.5 (b–e) from *a^t^/a^t^* or *R26R*/+; *Tg.Hoxb6-Cre*/+ mice (the latter were stained with X-Gal), as described in Materials and Methods. For purposes of comparison, images were proportionally scaled. The boundary of X-Gal staining marks dermis derived from lateral plate versus dermis derived from mesoderm (the lateral somitic frontier) and lies more dorsal than the *a^t^/a^t^* pigmentation boundary. (B) Quantitation of mean (± SEM) dorsal pigmentation area (*n* = 5) and somite-derived dermis area (*n* = 3) shows a significant difference (*p* < 0.005, *t*-test). (C) RNA in situ hybridization showing that *Tbx15* expression at E11.5 is complementary to *En1* expression on the flank (scale bars = 200 μm). The arrow indicates the boundary between the expression domains of the two genes.

To examine directly the relationship between the pigmentation boundary and dermis derived from lateral plate mesoderm, we made use of a *Cre* transgene driven by the *Hoxb6* promoter that was developed by Kuehn and colleagues (Lowe et al. 2000). As described by Lowe et al. (2000), midgestation embryos carrying both the *Hoxb6-Cre* transgene and the *R26R lacZ* reporter gene (Soriano 1999) exhibit X-Gal staining in lateral plate mesoderm but not somite-derived mesoderm of the trunk. In whole-mount skin preparations from P1.5 or P4.5 neonatal animals, we observed a ventral band of dark X-Gal staining corresponding to lateral plate-derived dermis, which represents 63% of the total circumference (Figure 8A). However, in parallel preparations from *a^t^*/*a^t^* mice, the ventral pheomelanin domain represents 47% of the total skin circumference; therefore, the proportions of total skin circumference occupied by dorsal eumelanin and somite-derived dermis are 53% and 37%, respectively (Figure 8B). These results indicate that the pigmentation boundary is clearly distinct from, and more ventral to, the boundary between lateral plate- and somite-derived dermis.

Because the pigmentation boundary lies in register with the limb–body wall junction (see Figure 2), we wondered whether mechanisms used for dorsoventral limb patterning might be related to those used to establish the pigmentation boundary. In the developing limb, *Engrailed1* (*En1*), *Wnt7a*, and *Lmx1b* are part of a network whose restricted domains of expression help to establish dorsoventral identity (reviewed in Niswander 2003). *En1* is transiently expressed in the developing flank; at E11.5, transverse abdominal sections reveal domains in the neural tube, somite-derived mesenchyme, and the ventral body wall (Figure 8C). An adjacent section hybridized with *Tbx15* reveals a complementary pattern in the flank, which provides additional evidence for developmental mechanisms that establish a pigmentation boundary entirely within lateral plate mesoderm and independent of lineage restrictions imposed by the lateral somitic frontier.

## Discussion

Several mutations and genes have been identified that affect the pattern of hair follicle development, but *Tbx15* is the only gene of which we are aware that affects the pattern of hair pigmentation in different body regions. Ventral areas that normally produce yellow hair in the trunk, limbs, and craniofacial regions are expanded in *de^H^*/*de^H^* mice and, in the trunk at least, represent inappropriate dorsal expression of an *Agouti* mRNA isoform that is normally restricted to ventral skin. The *de^H^* allele is caused by a large deletion that removes most of the *Tbx15* coding sequence, but the pleiotropic phenotype is caused by a simple loss of function for *Tbx15* rather than a dominant-negative or contiguous gene effect. In particular, there is no heterozygous phenotype, no other genes lie within or close to the deletion breakpoints, and the expression pattern of *Tbx15* is consistent with the spectrum of phenotypic abnormalities in both the original *de* allele and the *de^H^* allele. Finally, a *Tbx15* targeted allele has the same phenotype as *de^H^*.

Our results suggest that patterned expression of *Tbx15* provides an instructional cue required to establish the future identity of dorsal dermis with regard to pigmentary and hair length patterning. The ventral edge of *Tbx15* expression in the developing flank does not correspond to a known lineage compartment, but, like limb development, occurs within lateral plate mesoderm. These findings represent a novel role for T-box gene action in embryonic development and provide evidence for a previously unappreciated complexity to acquisition of dorsoventral positional identity in mammalian skin.

### Distinct Morphologic Regions Represent the Sum of Different Gradients

**Figure 9:**
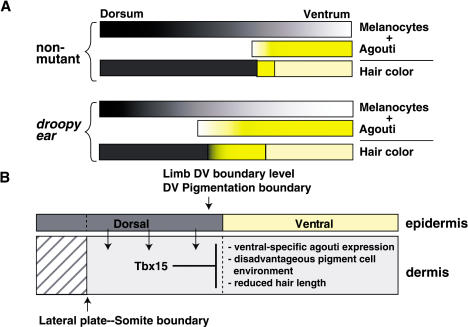
Model for Acquisition of Dorsoventral Patterning in the Trunk and the Role of *Tbx15* (A) A tricolor pigmentation pattern is generated by the combination of distinct mechanisms that affect distribution of *Agouti* mRNA and histochemical staining for melanocytes; effects of the latter mechanism by itself are evident in *a^e^*/*a^e^* mice (see Figure 1). In *a^t^/a^t^* mice, reduced hair melanocyte activity and high levels of *Agouti* mRNA in the ventrum lead to a cream color; as melanocyte activity gradually increases towards the dorsum, a lateral stripe is apparent on the flank. The distributions of *Agouti* mRNA and histochemical staining for melanocytes are both affected by *Tbx15* and are externally evident by a widening of the lateral stripe and an increased proportion of total skin occupied by the cream-colored area. (B) The lateral yellow stripe in *a^t^/a^t^* mice lies at the same level as the limb dorsoventral boundary. As described in the text, we propose that distinct dorsoventral compartments in ectoderm of the trunk provide an instructional cue to the mesoderm, leading to expression of *Tbx15* in dorsal trunk mesenchyme and acquisition of dorsal dermis character. In the absence of *Tbx15*, dorsal mesenchyme assumes ventral characteristics instead.

The visual boundary between dorsal and ventral skin in *a^t^*/*a^t^* mice is reminiscent of other systems in which adjacent compartments enforce a binary choice between alternative patterns of gene expression and cell fate (reviewed in Dahmann and Basler 1999). However, *Agouti* mRNA in both embryonic and postnatal skin is distributed along a gradient whose dorsal boundary is indistinct and overlaps with two additional gradients recognized by their effects on hair length and histochemical staining for melanocytes. The three gradients are close but not congruent, and it is their proximity that gives rise to the superficial distinction between dorsal and ventral skin of *a^t^*/*a^t^* mice. Indeed, slight differences between the regions of transition for pigment-type switching and pigment content give rise to a subtle yellow stripe along the flank (see Figures 1, 2, and 9A). Levels of *Agouti* mRNA remain high throughout the entire ventrum, but hair pigment content is reduced, giving rise to a cream-colored region in the ventrum that, depending on age and genetic backgrounds, may appear more or less distinct from the yellow flank stripe.

Loss of *Tbx15* affects dorsoventral transitions of hair length, pigment content, and expression of the ventral-specific *Agouti* isoform; however, the former two effects are subtle and contribute little, if at all, to the abnormal pigmentation of adult *de^H^*/*de^H^* mice. Thus, despite the abnormal pattern of dark skin in neonatal *de^H^*/*de^H^* mice (e.g., Figure 2D), the most obvious feature in adults is dorsal displacement of the “boundary” between black and yellow hair (Figure 9A).

### Genetics of *Tbx15*

Named for the presence of a DNA-binding domain first identified in the mouse *Brachyury* gene (haploinsufficiency causes a short tail), T box–containing genes have been identified as developmental regulators in a wide spectrum of tissues and multicellular organisms (reviewed in Papaioannou 2001). The *Tbx15* subfamily, which also includes *Tbx18* and *Tbx22*, is likely to have arisen during early chordate evolution since there is a single gene in amphioxus but no obvious homolog in the fly genome (Ruvinsky et al. 2000). Consistent with this relationship, the three genes are expressed in partially overlapping patterns that include anterior somites (*Tbx18* and *Tbx22*), limb mesenchyme (*Tbx15* and *Tbx18*), and craniofacial mesenchyme (all three genes, *Tbx15* more broadly than *Tbx18* or *Tbx22*) (Agulnik et al. 1998; Kraus et al. 2001; Braybrook et al. 2002; Bush et al. 2002; Herr et al. 2003). These observations suggest that an ancestral gene for *Tbx15*, *Tbx18*, and *Tbx22* may have been important for craniofacial development in cephalochordates, with acquisition of additional expression patterns and developmental functions in the limb and the trunk during early vertebrate evolution. Expression of *Tbx18* and *Tbx22* has not been reported in embryonic flank mesenchyme, which suggests that *Tbx15* is the only family member involved in establishing the dorsoventral identity of the trunk. However, it would not be surprising to find some degree of functional redundancy in animals mutated for two or three of the subfamily members in other body regions, particularly the limbs and the head. For example, mutations in *Tbx22* cause the human syndrome X-linked cleft palate and ankyloglossia (Braybrook et al. 2001). Despite high levels of *Tbx22* expression in periocular embryonic mesenchyme (Braybrook et al. 2002; Bush et al. 2002; Herr et al. 2003), the condition does not affect the eye, perhaps because residual activity is provided by *Tbx15* in the same region.

In an initial description of the expression and map location of mouse *Tbx15*, Agulnik et al. (1998) suggested human *Tbx15* that lies on Chromosome 1p11.1 as a candidate for acromegaloid facial appearance (AFA) syndrome, for which there is a weak positive LOD score to Chromosome 1p (Hughes et al. 1985). Originally described as a rare autosomal-dominant syndrome with progressive facial coarsening, overgrowth of the intraoral mucosa, and large, doughy hands, more recent case reports describe macrosomia, macrocephaly, or both and generalized hypertrichosis with progressive coarsening (Dallapiccola et al. 1992; Irvine et al. 1996; da Silva et al. 1998; Zelante et al. 2000). The *de^H^* phenotype exhibits little overlap with these features; instead, we suggest a more likely candidate for mutations of human *TBX15* would be frontofacionasal syndrome, an unmapped autosomal recessive condition characterized by brachycephaly, blepharophimosis, and midface hypoplasia (Reardon et al. 1994).

Two of us (S. Kuijper and F. Meijlink) became interested in the *de^H^* mutation because of its effects on skeletal development (Curry 1959) and the possibility that the *aristaless*-related gene *Alx3* might be allelic with *droopy ear* (ten Berge et al. 1998). In spite of similarities between skeletal phenotypes of *de^H^* and *Alx3* or *Alx4* mutants, subsequent experiments (unpublished data) excluded allelism of *Alx3* and *de^H^*, and a full description of the *Tbx15* skeletal phenotype will be published elsewhere.

### Developmental Mechanism of *Tbx15* Expression and Action in the Skin

Our attention to the role of *Tbx15* in pigment patterning was motivated by the effects of *Agouti* in postnatal animals. However, *Agouti* is also expressed in the embryo, where it provides a convenient marker of ventral dermis identity. Because an expanded domain of embryonic *Agouti* expression in *de^H^*/*de^H^* animals is detectable by E14.5, the effects of *Tbx15* on dorsoventral patterning must occur prior to this time. Among other T-box genes whose developmental actions are at least partially understood, two general themes have emerged, one focused on the ability to specify alternative fates for an undifferentiated group of precursor cells and another focused on the ability to support proliferative expansion of a cell population whose fate is already determined (reviewed in Tada and Smith 2001). Either mechanism may apply to the apparent dorsal-to-ventral transformation in *de^H^*/*de^H^* mice. For example, while the expanded domain of *Agouti* expression in postnatal *de^H^*/*de^H^* animals can be traced to events that occur between E11.5 and E13.5, the underlying cause may be that embryonic cells in dorsolateral mesenchyme acquire a ventral rather than dorsal identity or that those cells fail to proliferate normally, followed by compensatory expansion of ventral cells. Cell lineage studies should provide a definitive answer, but we favor the latter hypothesis, because measurements of dorsoventral regions according to hair color in *de^H^*/*de^H^* mice revealed a small increase of the cream-colored ventral region in addition to the approximate doubling of the yellow flank region (see Figure 2).

In embryonic mesenchyme, expression of *Tbx15* and *Agouti* are complementary, and it is possible that *Tbx15* acts directly to inhibit *Agouti* transcription in dorsolateral mesenchyme. However, the ability of *Tbx15* to suppress expression of the ventral-specific *Agouti* isoform in postnatal mice is likely to be indirect, since postnatal expression of *Tbx15* occurs broadly along the dorsoventral axis and overlaps extensively with that of *Agouti*. In either case, the targets of *Tbx15* action in the skin include genes in addition to *Agouti*, since hair length and melanocyte distribution exhibit a demonstrable, albeit subtle, alteration in animals that carry a null *Agouti* allele. One potential target is *Alx4*, which, like *Agouti*, is expressed in ventral embryonic mesenchyme, and, when mutated, affects hair-follicle as well as limb and craniofacial development (Qu et al. 1997, 1998; Wu et al. 2000; Wuyts et al. 2000; Mavrogiannis et al. 2001). However, expression of ventral markers such as *Alx4*, as well as *Alx3* and *Msx2*, appears to be unaffected at E11.5 in *de^H^*/*de^H^* embryos (data not shown).

### Differences and Similarities to Dorsoventral Limb Patterning

Loss of *Tbx15* also affects regional distribution of hair color in the limbs, with areas that would normally produce black hair giving rise to yellow hair instead. However, neither normal patterns of pigment-type synthesis in the limb nor their disruption in *de^H^*/*de^H^* mice correspond to obvious developmental compartments. Furthermore, losses of function for *En1* or *Wnt7a*, which cause a partial transformation of the distal limb from dorsum to ventrum (Loomis et al. 1996) or ventrum to dorsum (Parr and McMahon 1995), respectively, have no effect on regional patterns of *Agouti* expression or distribution of hair-color regions (Y. Chen, unpublished data). (Ectopic pigmentation of the ventral footpads that develops in *En1* mutant mice is unrelated to pigment-type synthesis and instead likely reflects a requirement for *En1*, independent of *Wnt7a*, to repress migration or proliferation (or both) of pigment cells in ventral epidermis [Cygan et al. 1997; Loomis et al. 1998].)

These considerations notwithstanding, control of dorsoventral trunk pattern by *Tbx15* shares certain features with control of dorsoventral limb patterning by *Lmx1b*, a LIM domain transcription factor that acts downstream of *Wnt7a* and *En1* (Riddle et al. 1995; Vogel et al. 1995; Cygan et al. 1997; Logan et al. 1997; Loomis et al. 1998; Chen and Johnson 2002). Both *Tbx15* and *Lmx1b* act autonomously in mesenchymal cells to promote a dorsal identity, yet have expression domains that do not correspond to cell lineage compartments in the flank (*Tbx15*) or the limb (*Lmx1b*) (Altabef et al. 1997; Michaud et al. 1997). In the case of *Lmx1b*, its expression in the distal limb depends on *Wnt7a* produced in the overlying dorsal ectoderm (Riddle et al. 1995; Cygan et al. 1997; Loomis et al. 1998). *Wnt7a*, in turn, is restricted to dorsal ectoderm by *En1* in the ventral ectoderm (Loomis et al. 1996; Cygan et al. 1997; Logan et al. 1997), whose expression marks a lineage boundary coincident with the dorsoventral midline of the apical ectodermal ridge (Altabef et al. 1997; Michaud et al. 1997; Kimmel et al. 2000). As described above, *En1* or *Wnt7a* mutations have not been reported to affect patterns of hair-color distribution (C. Loomis, personal communication; Parr and McMahon 1995; Loomis et al. 1996). However, the essential theme that ectodermal lineage compartments control the fate of underlying mesenchyme in developing limbs may apply to the trunk as well as the limb. The mammary glands also develop at a stereotyped dorsoventral position and depend on epithelial–mesenchymal interactions. However, the number and apparent position of the mammary glands are normal in *de^H^*/*de^H^* animals, indicating the existence of additional mechanisms that control dorsoventral patterning in the trunk as well as in the limbs.

These ideas are summarized in the model shown in Figure 9B. We speculate that a diffusible signal from dorsal trunk ectoderm, at or prior to E11.5, promotes expression of *Tbx15* in dorsal trunk mesenchyme, which then establishes dorsal positional identity of those cells as manifested by differences in *Agouti* expression, pigment-cell development, and hair growth. Because the ventral limit of *Tbx15* expression corresponds to the dorsal limit of *En1* expression and because the normal position of the pigmentation boundary lies approximately in register with the limb-bud outgrowths, we depict the position of a putative dorsoventral boundary in trunk ectoderm as coincident with the limb dorsoventral boundary. This model is consistent with studies in the chick, where distinct dorsal and ventral lineage compartments exist for ectoderm in both the limb (Altabef et al. 1997, 2000; Michaud et al. 1997; Kimmel et al. 2000) and interlimb regions (Altabef et al. 1997, 2000), but not for limb mesoderm (Altabef et al. 1997; Michaud et al. 1997). In fact, the same mechanism that determines dorsoventral position of the limbs and the apical ectodermal ridge may also act on expression of *Tbx15* in the trunk, since ectopic limbs induced in the interlimb region by application of FGF beads develop along a single line that is coincident with normal limb buds (and the future pigmentation boundary) (Cohn et al. 1995; Crossley et al. 1996; Vogel et al. 1996; Altabef et al. 1997, 2000).

Our model predicts that ectopic expression of *Tbx15* in ventral mesenchyme should give rise to a dorsalized pigmentation phenotype and could be tested with gain-of-function approaches. However, *Tbx15* expression is very dynamic and is restricted to dorsal mesoderm only from E11.5 to E13.5. It is possible that *Tbx15* influences skin patterning in a very narrow window of development; alternatively, establishment of dorsal identity by *Tbx15* may require another as-yet-unidentified factor that is only present in the mesenchyme underlying dorsal ectoderm.

### Pigmentation Patterns and *Tbx15* in Other Mammals

The lateral somitic frontier, defined as the lineage boundary between somite-derived versus lateral plate-derived mesoderm, is established during somitogenesis early in development (Mauger 1972; Christ et al. 1983; Olivera-Martinez et al. 2000; Nowicki et al. 2003), but remains distinct in postnatal animals despite the potential for extensive cell mixing (see Figure 8). However, our transplantation and fate-mapping studies demonstrate that the lateral somitic frontier lies dorsal to the pigmentation boundary and does not obviously correlate with a difference in skin morphology. An additional dorsoventral domain that is not externally apparent has emerged from studies of *Msx1*, whose expression marks a subgroup of somite-derived mesenchymal cells that contribute to dermis in a narrow stripe along the paraspinal region (Houzelstein et al. 2000). Thus, there exist at least three distinct boundaries in postnatal mammalian skin that are parallel to the sagittal plane, marked by differences in pigment-type synthesis, differences in cell lineage, and differences in expression of *Msx1*.

In rodents, only the pigmentation boundary is evident externally, but many mammals have more complicated patterns of hair type, length, and/or color that vary along the dorsoventral axis. Raccoons, squirrels, skunks, and many different ungulates exhibit lateral stripes whose developmental origins have not been investigated, but may correspond to the lateral somitic frontier, the paraspinal *Msx1* compartment, or an interaction between these domains.

The effect of *Tbx15* on pigmentation in laboratory mice is reminiscent of coat-color patterns in both selected and natural populations of other mammals. Saddle markings are common in some dog breeds, such as German shepherds, and in certain populations of Peromyscus polionotus, in which a dorsal extension of ventral depigmentation provides an adaptive advantage to subspecies that live on white sand reefs (Blair 1951; Kaufman 1974; Belk and Smith 1996). Neither German shepherds nor deer mice have craniofacial characteristics similar to the *de^H^* mutation, but the pigmentation patterns in these animals could represent alterations in the regulation or action of *Tbx15* activity. From the opposite perspective, the effects of *Tbx15* on coat color are only apparent in certain genetic backgrounds and may not be evident at all in mammals that lack dorsoventral pigmentation patterns. Studying the sequence and expression of *Tbx15* in other vertebrates may provide additional insight into patterns that affect the skeleton as well as the pigmentary system.

## Materials and Methods

### 

#### Mice

All mice were obtained originally from The Jackson Laboratory (Bar Harbor, Maine, United States), except the BA strain (Stanford Veterinary Services Center, Stanford, California, United States), *Hoxb6-Cre* transgenic mice (kindly provided by M. Kuehn of the National Institutes of Health, Bethesda, Maryland, United States), mice carrying the *R26R lacZ* reporter allele (kindly provided by P. Soriano, Fred Hutchinson Cancer Research Center, Seattle, Washington, United States), and C57BL/6J (B6) *a^e^/a^e^* mice (kindly provided by L. Siracusa, Jefferson Medical College, Philadelphia, Pennsylvania, United States). The *de^H^* mutation arose in the 1960s in Harwell, probably on the BN strain background (C. Beechey, personal communication). We obtained *de^H^* on a B6/EiC3H background, introduced the *a^t^* allele from the BTBR strain, and have maintained the line as a mixed *de^H^*/+ × *de^H^*/+ intercross stock with periodic outcrossing to BTBR or B6. For timed matings, the morning of the plug was considered E0.5. Postnatally, the day of birth was considered to be P0.5.

#### Phenotypic analysis

For measurements of hair length and color, the entire interlimb region of skin was first dissected with a single incision at the dorsal midline and preserved with powdered sodium bicarbonate. Slices 2–2.5 mm in width were then prepared parallel to the dorsoventral axis, hair length boundaries determined from electronic images with Adobe Photoshop (San Jose, California, United States), and measurements obtained using ImageJ (National Institutes of Health). This approach samples awls and auchenes, because they are much thicker and therefore visually more predominant than zigzag underhairs. To assess dorsoventral variation in hair-type distribution, several hundred hairs were plucked from the middorsum or midventrum of 8-wk-old male BA strain animals, then sorted and categorized using a dissection microscope. No attempt was made to distinguish between awls and auchenes.

For skin histology, 12 μm sections from paraffin-embedded tissue were stained with hematoxylin and eosin. For DOPA staining, the dermis and epidermis were split after 3 h of incubation in 2 M sodium bromide at 37°C (this preparation causes most hair follicles to remain with the dermis), individually fixed for 1 h, then rinsed and stained with 0.1% L-DOPA (Sigma, St. Louis, Missouri, United States), 0.1 M sodium phosphate buffer (pH 6.8) for 5 h at 37°C in the dark, changing the staining solution after 1 h. The samples were then fixed overnight, dehydrated, and mounted. This method is sufficient to stain interfollicular melanocytes without creating a high background. The fixative used was always 4% paraformaldehyde.

#### Positional cloning

A high-resolution map for *de^H^* was generated from an intersubspecific intercross between *de^H^*/*de^H^* and CAST/Ei mice. We initially localized *de^H^* to a 1 cM interval between D3Mit233 and D3Mit11. F_2_ animals carrying recombinant chromosomes between these markers whose genotype at *de* was indeterminate (*de^H^*/+ or +/+) were progeny-tested by crossing to *de^H^*/*de^H^* animals. Further genetic mapping established a minimal region of 0.1 cM between D3Mit213 and 16.MMHAP32FLF1; these markers were used to initiate construction of a physical map with BAC genomic clones (Research Genetics, Huntsville, Alabama, United States, and Genome Systems, St. Louis, Missouri, United States). End sequence from those BACs was used to develop SSCP markers M1 to M3, as depicted in Figure 3, and to establish a minimal physical interval of 1.4 Mb. Primer pairs used were TTCCCTCCAATAAGTTCTGGGTACC and AAGCTTGCTGCTCTGGATTCCATTTGTAG for M1, CCTTCATTTTTTTTTCAAGTAAAA and AAGCTTGGCTTAGTCCCAGTGGC for M2, CCTCCAGGAAGATCTACTAGGCAC and ATGGAAAAAAAAAAGTAAGATTGAAAG for M3, and TGGTTATCGATCTGTGGACCATTC and AAGTGAGAGAGCAGGATGGACCAC for M4 (the M4 marker represents STS 16.MMHAP32FLF1). Genomic sequence and annotations were obtained from the UCSC Genome Browser February 2003 assembly version mm3 (http://genome.ucsc.edu); the 1.4 Mb interval between M1 and M4 contains eight genes: four hydroxysteroid dehydrogenase isomerases, *Hsd3b3, Hsd3b2*, *Hsd3b6*, and *Hsd3b1*; an hydroacid oxidase, *Hao3*; a tryptophanyl-tRNA synthetase, *Wars2*; a T-box gene, *Tbx15*; and a novel gene, *4931427F14Rik*. In the genome sequence, M1 primers correspond to AGGCCTCCAATAAGTTCTGGGTACC and AAGCTTGCTCTCTGGATTCCATTTGTAG, the M2 reverse primer corresponds to AAGCTTGGCTTTAGTCCCAGTGGGC, and the M3 primers correspond to CCTCCAGGAAGAATCTACTAGGCAC and AATGAAAAAAAAAAAAGTAAGATTGAAAG. Minor differences among the sequences of the primers we obtained from the BAC ends and the public genome sequence may represent strain differences or sequencing errors on the BAC DNA.

A multiplex genotyping assay was developed to genotype for the *de^H^* deletion using primers GGAGCAGATCCAATTGCTTT, TCCATAGCCCATCTTCACAA, and CATGTCCACTTCTGCTTCCA. This PCR assay produces a 392 bp product from the *de^H^* chromosome and a 595 bp product from the nonmutant chromosome.

#### Gene targeting

A targeted allele of *Tbx15* was constructed using the same approach described in Russ et al. (2000). In brief, an *IRES-LacZ-neo* cassette with 5′ and 3′ homology arms of 3.5 kb and 1.8 kb was inserted into a unique BamHI site that lies 479 nucleotides downstream of the transcriptional initiation site (relative to the mRNA sequence) in exon 3. Positive ES clones were injected into B6 blastocysts, and chimeric founders crossed to either B6 mice or to *de^H^*/+ animals.

#### In situ hybridization

In situ hybridization was carried out on 12-μm paraffin sections using digoxigenin-labeled RNA probes (Roche Diagnostics, Indianapolis, Indiana, United States) according to standard protocols (Wilkinson and Nieto 1993). Embryos and postnatal skin samples were obtained from intercrosses of *de^H^*/*+* mice. Embryos E13.5 or younger were fixed for 24 h; those older than E13.5 and postnatal skin were fixed for 36–48 h prior to embedding. The *Tbx15* probe was generated by RT–PCR using primers GGCGGCTAAAATGAGTGAAC and TGCCTGCTTTGGTGATGAT (corresponds to exons 1 and 2), and the *En1* probe was generated by PCR from genomic DNA using primers ACGCACCAGGAAGCTAAAGA and AGCAACGAAAACGAAACTGG (located in the last exon). The *Agouti* probe corresponds to the protein-coding sequence.

#### Embryonic skin transplantation

(BTBR-*a^t^*/*a^t^* × B6-*a*/*a*)F_1_ embryos at E12.5 were dissected in sterile Tyrode's solution, and embryonic skin was divided into dorsal, flank, and ventral pieces, each 1–2 mm^2^ in size, as shown in Figure 7. Skin fragments were grafted to the testes of congenic animals as follows. After anesthetization with 2.5% Avertin, a 1.5-cm incision in the skin and body wall was made at a point level with the top of the limbs. The fat pads were pulled out and laid on the outside of the body, exposing the testes. Forceps were used to introduce a small hole in the testis capsule through which a piece of dissected embryonic skin was inserted, the testes were then replaced into the abdominal cavity, and the wound was closed in both the body wall and the skin. After 21 days, mice that received grafts were sacrificed and the resulting hair was dissected from the testes and examined.

#### Fate-mapping the lateral somitic frontier

The *Hoxb6-Cre* transgene described by Kuehn and colleagues (Lowe et al. 2000) is expressed in the lateral plate but not the somitic mesoderm of the trunk, beginning at E9.5. Animals doubly heterozygous for this transgene and the *R26R* reporter gene were used as a source of whole skin at P1.5 or P4.5. Skin sections parallel to the dorsoventral axis were prepared with a single incision along the ventral midline and stained for β-galactosidase activity using standard protocols at room temperature. The P1.5 sample was stained overnight and the P4.5 samples were stained for 5.5 h. Similar nonstained skin sections were prepared from animals carrying the *a^t^* allele. Images of the different skin fragments were aligned and scaled, and the relative position of the somite–lateral plate and the pigmentation boundaries were measured using ImageJ.

## Supporting Information

The GenBank (http://www.ncbi.nlm.nih.gov/Genbank/index.html) accession numbers discussed in this paper are for *4931427F14Rik* (AK016477), *Agouti* gene (L06451), *Alx3* (U96109), *Alx4* (AF001465), *En1*(L12703), *M6pr-ps* (X64069), *Tbx14* (AF013282), *Tbx15* (AF041822), *Tbx18* (AF306666), and *Tbx22* (NM_145224).

The OMIM (http://www.ncbi.nlm.nih.gov/omim/) accession numbers discussed in this paper are for acromegaloid facial appearance (MIM 102150), frontofacionasal syndrome (MIM 229400) and human syndrome X-linked cleft palate and ankyloglossia (MIM 303400).

## Abbreviations

*a^e^*: *extreme nonagouti* allele
AFA: acromegaloid facial appearance
*a^t^*: *black-and-tan* allele
*A^W^*: *white-bellied Agouti* allele
B6: C57BL/6J mice
BAC: bacterial artificial chromosome
*de^H^*: *droopy ear* allele that arose in Harwell
E10.5: embryonic day 10.5
*En1*: *Engrailed1*
*M6pr-ps*: mannose-6-phosphate receptor pseudogene
P4.5: postnatal day 4.5
*Tbx15*: T-box gene 15
